# Involvement of alcohol in injury cases in rural Sri Lanka: prevalence and associated factors among in-patients in three primary care hospitals

**DOI:** 10.1186/s12889-022-12958-8

**Published:** 2022-03-16

**Authors:** L. Schölin, M. Weerasinghe, S. Agampodi, U. Chathurange, S. Rajapaksha, A. Holloway, J. Norrie, F. Mohamed, M. Eddleston, M. Pearson

**Affiliations:** 1 Centre for Pesticide Suicide Prevention & Centre for Cardiovascular Science, University of Edinburgh, Edinburgh, UK; 2 Department of Community Medicine, Faculty of Medicine & Allied Sciences, Rajarata University of Sri Lanka, Anuradhapura, Sri Lanka; 3 South Asian Clinical Toxicology Research Collaboration, Faculty of Medicine, University of Peradeniya, Peradeniya, Sri Lanka; 4 Nursing Studies, School of Health in Social Science, University of Edinburgh, Edinburgh, UK; 5 Usher Institute, Edinburgh Clinical Trials Unit, University of Edinburgh, Edinburgh, UK

**Keywords:** Alcohol-related injuries, Sri Lanka, Blood alcohol concentration, AUDIT

## Abstract

**Background:**

Injuries account for a major proportion of global morbidity and mortality related to alcohol use. Information on the prevalence of alcohol-related injury in rural Sri Lanka is limited. The aims of this study were to determine the burden of alcohol-related injury in a hospital-based sample in rural Sri Lanka and explore factors associated with an increased risk of alcohol-related injury.

**Methods:**

Involvement of alcohol in injury amongst in-patients was assessed in three hospitals in the North Central Province of Sri Lanka over 6 months. Adult (≥ 18 years) patients were eligible. Patients were assessed for: injury characteristics, current alcohol use (in the past year) using the Alcohol Use Disorder Identification Test (AUDIT), and acute intoxication. Patients with a blood alcohol concentration (BAC) reading equivalent of 10 mg/dL (2.17 mmol/L) were considered as having an alcohol-related injury. Binary logistic regression was used to explore association between alcohol-related injury and demographic and injury characteristics.

**Results:**

A total of 883 injured patients were eligible and consented to the study. No alcohol use was reported by 487 (55.2%) of patients (35.6% of men, 95.2% of women). Prevalence of alcohol-related injuries was 14.8% overall and 32.8% among current alcohol users. Almost all patients with an alcohol-related injury were male (122/123; 99.2%); 24 (18.8%) of these patients scored positive for possible alcohol dependence. Patients with an alcohol-related injury had significantly higher AUDIT scores (median = 15 vs 6, *p* < 0.001), were significantly more likely to be aged 26–40 (OR 2.29, 95% CI:1.11, 4.72) or 41–55 years (OR 2.76, 95% CI: 1.29, 5.90) (compared to 18–25 years), to have a transport-related injury (OR 5.14, 95% CI: 2.30, 11.49) (compared to animal/plant sting/bite), and have intentional injuries (OR 3.47, 95% CI: 1.01, 11.87).

**Conclusions:**

One in three injuries among people who drank alcohol in this sample were alcohol-related. In addition, problematic alcohol use was higher among those with alcohol-related injury. Further work is needed to explore whether this prevalence of alcohol-related injury is reflected in other rural settings in Sri Lanka.

**Supplementary Information:**

The online version contains supplementary material available at 10.1186/s12889-022-12958-8.

## Introduction

Alcohol is a major global health problem, contributing to 1 in 20 deaths globally [1]. In 2016, an estimated 36.8 deaths per 100 000 population were attributed to alcohol in the South-East Asia region, despite a regional lifetime abstention rate of 56.6% [1]. Per capita consumption in the region is 4.5 L/year overall and 9.8 L among drinkers, compared to a global average of 13.9 L [1].

Sri Lanka has a major problem with alcohol consumption and disease, which has negative impact economically [2] as well as socially [3]. Per capita consumption is low at 4.3 L/year overall, as only 28.7% report consuming alcohol in the last year [1]. Current drinking (within the last 6 months) has been reported as 48.1% among males and 1.2% among females, with higher prevalence in urban areas [4]. Per capita consumption in drinkers in 2016 was estimated at 14.9 L, compared to the global average of 15.1 L. Consumption among Sri Lankan drinkers is higher in men than women (18.9 and 6.7 L, respectively) [1]. In men, hazardous drinking is associated with a low level of education, while current drinking is associated with middle level education (6–11 years of fulltime education) and high income [4]. Consumption in heavy episodic patterns is common: 2016 data from WHO shows that 31.7% of drinkers consumed > 60 g of alcohol on one occasion at least once in the last month, with a prevalence of 40.8% and 13.2% in male and female drinkers, respectively [1].

Alcohol consumption in Sri Lanka is predominantly of spirits, with approximately 40% of all alcohol consumed being illicit [1]. Illicit alcohol is a particular issue due to health issues resulting from its toxicity [5], as well as the tendency for it to be consumed in large quantities due to accessibility and low price [6]. Research undertaken in rural villages in the North Central Province, the majority of alcohol consumed by older men is locally produced illicit alcohol (commonly in the form of Kassipu) while younger men preferred commercially produced non-illicit products [7].

Despite a high level of abstention, there is a significant burden of alcohol-related harm in Sri Lanka. In 2016, alcohol-attributable deaths per 100,000 population were 2,880 for liver cirrhosis, 675 for road traffic injuries, and 649 for cancer [1]. Alcohol has been identified as a causal contributing factor in the high incidence of deliberate self-harm, suicide, domestic violence, poverty, malnutrition, and traffic injuries in rural Sri Lanka [3, 8–11].

Alcohol-related injuries are a leading cause of death and disease in lower-middle income countries (LMIC) [1]. In Sri Lanka, overall injury rates are high, with annual mortality and disability rates of 177 and 290 per 100,000 population [12]. Traumatic injuries have been the leading cause of hospital admission in Sri Lanka and among the top ten causes of hospital deaths for the last two decades [13].

Alcohol use increases the risk of injury, in particular traffic accidents, which show a causal relationship between alcohol consumption and injury risk [14, 15]. Alcohol-related injury risk, however, depends on context, including overall alcohol policy strictness, drinking patterns, and type of injury. A study from 22 countries found higher levels of alcohol-related injuries in countries with high levels of detrimental drinking patterns (defined through country-level data on factors including heavy drinking episodes, level of abstinence, drinking with meals, and drinking in public places, see Rehm et al. [16]) and/or few policy restrictions on alcohol use [17]. International research, involving 44 emergency departments (EDs) from 28 countries, found that the average prevalence of injuries where patients had a blood alcohol concentration of 10 mg/dL (defined as alcohol-related) was 24%, ranging from 4%–46% among all patients and 4%–80% in current drinkers [18].

The prevalence of recent alcohol use in patients presenting to hospital in Sri Lanka is unknown due to a lack of hospital-based injury studies in which both acute and current use of alcohol is formally assessed. The aims of this study were to a) estimate the burden of alcohol-related injury in a hospital-based sample of injury patients admitted to hospital in Sri Lanka’s North Central Province, and b) explore factors associated with an increased risk of having an alcohol-related injury.

## Materials and methods

### Setting

The study was based in three hospitals representing different levels of primary and secondary care hospitals in Sri Lanka’s North Central Province, a predominant agricultural area: District Base Hospital (DBH) Thambuttegama, District Hospital (DH) Rajanganaya Tract 11, and DH Galnewa (Table 1). The hospitals were purposively selected to examine whether different injuries presented to the different levels of hospitals. DBH Thambuttegama receives both primary presentations and transfers from local hospitals while other two hospitals predominantly receive direct admissions.

**Table 1:** Characteristics of study hospitals

Hospital	Wards	Other units^a^	Beds	Staff
DBH Thambuttegama	7	14	165	299
DH Rajanganaya	4	13	82	44
DH Galnewa	3	5	31	24

^a^Includes: outpatient department, theatres, mortuary, injection room, labour room, medical and antenatal clinics, dressing room

**Fig. 1:**
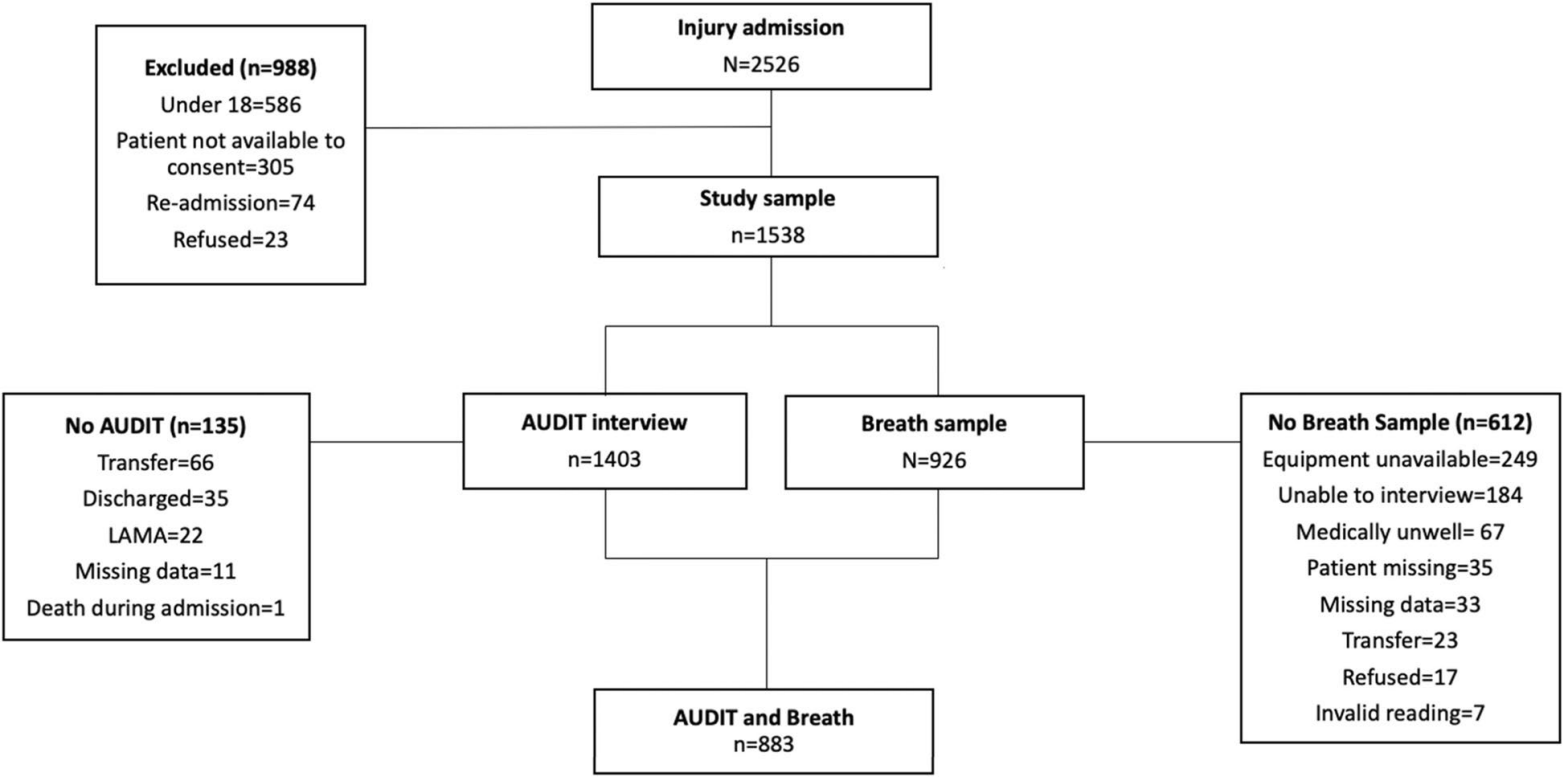
Sample flow chart

Adult patients (aged 18 and older) were admitted to an in-patient ward in one of the three study hospitals between May and November 2018 and able to provide consent were eligible for the study. We excluded patients with readmission of injuries “old wounds”, or under 18 years old, or unable to consent. We aimed to operationalise an inclusion criteria, with a cut-off point of 12 h between injury and the admission. However, time of injury was not routinely recorded and was missing for 66% of eligible in-patients. We were therefore unable to operationalise this cut-off. There was no significant difference in prevalence of patients who had a positive breathalyser test for whom the time of injury was known compared to those where the time was unknown (12.1% vs 15.7%, *p* = 0.091). The patient recruitment process is shown in Fig. 1. The study was approved by the Rajarata University of Sri Lanka Ethics Committee (ERC/2018/07).

### Data collection

All patients admitted to the wards who gave written consent (or whose relatives provided consent) were included in the study. Interviews were carried out on the wards; acutely ill patients were only considered for participation in the study when the medical staff advised they were fit for interview, or by obtaining proxy consent from their relatives. Research assistants (RAs) initially approached patients to obtain a breathalyser sample. In addition to a breathalyser sample, information about the injury and current alcohol use was collected using validated questionnaires (see Supplementary file 1). The RAs involved in the interviews had previous experience of community-based health research projects in the province and underwent extensive training and supervision. Routine checks of data quality and reliability were undertaken periodically during the study.

We were not able to collect information about drinking patterns according to the AUDIT or breathalyser sample for a significant number of patients (see Fig. 1), though obtaining a breath sample was more difficult. Significant numbers of injury admissions were transferred, discharged or left hospital before the RA was able to interview the patients. There was no significant difference in relation to age, gender or occupation for patients whom an AUDIT interview was obtained. The group of patients without a breathalyser sample were significantly older than those who provided a sample (median age 42.0 years vs 38.5 years, *p* = 0.002), had a lower proportion males (62.0% vs 68.3%, *p* = 0.009), and a higher proportion attendending Thambuttegama hospital (70.3% vs 63.5%, *p* = 0.019).

### Variables

#### Injury data

We interviewed patients or relatives based on an injury data collection tool modified from one developed by the Sri Lankan Ministry of Health. The tool included demographic data, information related to the injury (mechanism, place of occurrence, intent), affected body region, nature, evidence of alcohol and substance use, disability at the time of hospital discharge, and outcome (Supplementary file 1). In this paper we focus on injury mechanism, intent, and place and activity at the time of the injury in relation to alcohol use.

#### Acute alcohol use (alcohol-related injury)

Acute alcohol use was measured using a breathalyser (Drager Alcotest 6820) upon admission to the hospital. Physicians determined the suitability for taking the sample, and an adequate breath producing a valid concentration was required for the study. Breathalyser results equivalent to a blood alcohol concentration (BAC) of 10 mg/dL were considered positive, defining the injury as being alcohol-related. This sensitive definition, using a relatively low BAC, has been used previously [19]. Cherpitel et al. [20] in their analysis of data from the Emergency Room Collaborative Alcohol Analysis Project (ERCAAP) found that the “log odds of injury for those falling into each level of the range (0.01 mg/% [10 mg/dL] and 0.05 mg/%) was not found to increase linearly with BAC level relative to those with a BAC of 0, nor did the log odds of injury differ significantly for each categorized BAC level above 0.01.” (p.98) [20]. Using a binary variable with the cut-off point of 10 mg/dL (i.e. 0.01 mg/%, 2.17 mmol/L) was therefore considered appropriate. As there was insufficient information about the time of injury and time of breathalyser reading for a significant number of cases, we included all eligible cases despite time elapsed between injury and breathalyser sample being unknown.

#### Current alcohol use

A validated Sinhala version of the Alcohol User Disorder Identification Test (AUDIT) (Supplementary File 2) screening tool [21, 22] was used to collect a history of alcohol use in the past 12 months for each patient. Frequency of alcohol use includes response options ranging from never to four times per week or more. Patients who reported any drinking (AUDIT > 0) in the last year were considered current users. Median score using all ten items on the AUDIT questionnaires was calculated for the entire sample and for those who had a positive breathalyzer sample. The item for heavy episodic drinking was explored separately to provide details on the extent of drinking in heavy episodic patterns. Using the validated cut-off points for a Sri Lankan sample low-risk drinking was defined as a score of 0–7, hazardous drinking as 8–15, probable harmful use 16–19, and possible dependence ≥ 20 [21]. Heavy episodic drinking was ascertained by asking how often (ranging from never to daily or almost daily), in the past 12 months, the patient had consumed any of the following amounts of typical beverages consumed in Sri Lanka in one occasion: normal arrack (1/4 of a bottle), whisky (100 ml/2 glasses), Kasippu (illegal arrack) (> 1/2 of a bottle), beer (3–4 bottles), strong beer (2 bottles), toddy (2–3 bottles).

### Data analysis

Data was collected in a paper-based form and entered into Epiinfo. All subsequent analysis was performed in SPSS software version 25. Descriptive statistics were produced for demographic characteristics and injury characteristics by alcohol-related injury status (BAC 10 mg/dL positive/negative). Differences between groups were compared using a Chi-square test (*X*^2^) for all categorical variables. Medians and interquartile range (IQR) were calculated for continuous variables and differences between groups were analysed using Mann Whitney U test as the data was not normally distributed. Exploratory binary logistic regression analyses were conducted for each individual variable to assess which variables to include in a final logistic regression. The association between current drinking and an alcohol-related injury impacted on the model (Spearman *r* = 0.60, *p* < 0.001) and was therefore excluded. Furthermore, as only one woman had a positive breathalyser sample, we excluded gender from the final model. Two-sided *p* values were reported for each test and *p* < 0.05 was considered statistically significant.

## Results

In total, 2,526 patients were admitted for injury during the data collection period of which 1,538 were eligible and consented to take part in the study. Of these, 883 patients (57.4%) were able to provide a breathalyser sample and AUDIT interview, which forms the sample for this paper (Fig. 1). Demographic characteristics of the sample are outlined in Table 2, showed that most patients were male (67.2%), working as farmers (25.7%) or as daily wage laborers (16.1%), and admitted to Thambuttegama DBH (62.8%) (Table 2). The median age was 38 years (range = 18–83, IQR = 29–50). Current alcohol use (last 12 months) was reported by 396/833 (44.8%), more often by men (96.5%) than women (3.5%). The prevalence of having an alcohol-related injury (indicated by a positive breathalyser sample) in the total sample was 14.5% and 32.8% among current alcohol users.

**Table 2:** Characteristics of study population, *n* (%)

Characteristic	Category	Total (*n* = 883)	< 10 mg/dL (*n* = 755)	≥ 10 mg/dL (*n* = 128)	*X*^2^	*p*-value
Sex	Male	593 (67.2)	466 (61.7)	127 (99.2)	69.8	< *0.001*
	Female	290 (32.7)	289 (38.3)	1 (0.8)		
Age, median^a^ (IQR)	–	38 (29–50)	38 (28–50)	41.0 (34–51)		*0.047*
Age group	18–25	153 (17.3)	142(18.8)	11 (8.6)	12.3	*0.006*
	26–40	342 (38.7)	290 (38.4)	52 (40.6)		
	41–55	253 (28.7)	204 (27.0)	49 (38.3)		
	≥ 55	135 (15.3)	119 (15.8)	16 (12.5)		
Occupation	Farmer	227 (25.7)	181 (24.0)	46 (35.9)	51.1	< *0.001*
	Daily wage labour	142 (16.1)	105 (13.9)	37 (28.9)		
	Housewife	137 (15.5)	136 (18.0)	1 (0.8)		
	Other^b^	120 (13.3)	113 (15.0)	7 (5.5)		
	Government employee	59 (6.7)	51 (6.8)	8 (6.3)		
	Salaried employee	84 (9.5)	73 (9.7)	11 (8.6)		
	Self-employed	57 (6.5)	46 (6.1)	11 (8.6)		
	Business	57 (6.5)	50 (6.6)	7 (5.5)		
Hospital	Thambuttegama	556 (62.8)	474 (62.8)	79 (61.7)	1.9	0.375
	Galnewa	186 (21.0)	154 (20.4)	32 (25.0)		
	Rajanganaya	144 (16.3)	127 (16.8)	17 (13.3)		
Current drinker	Yes (AUDIT** ≥ **1)	396 (44.8)	270 (35.8)	126 (98.4)	173.8	< *0.001*
	No (AUDIT < 1)	487 (55.2)	485 (64.2)	2 (1.6)		

^a^Differences between groups compared with Mann Whitney U test; ^b^Includes: ‘other’, security forces, retired, army, foreign employed, garment worker. Due to small numbers in categories, all subsequent analysis merged businessman, housewife and government employed into the ‘other’ category

### Current alcohol use

Most patients were low risk drinkers (666/883), of whom 487 (55%) scored 0 and were defined as abstainers. More patients with alcohol-related injuries scored in the higher AUDIT risk categories than patients with injuries that were not alcohol-related and for 18.8% of those with positive BAC their AUDIT score indicated possible dependence compared to 1.3% of those with a BAC < 10 mg/dL (*p* < 0.001). Median AUDIT score among drinkers was 8.0 (IQR = 4–14), ranging from a score of 1 to 38. Median score was significantly higher for those with an alcohol-related injury (15, IQR = 11–19) than those who had a BAC < 10 mg/dL (6, IQR = 0–4) (*p* < 0.001). Two patients who tested positive for acute alcohol use reported being non-drinkers on the AUDIT. Among those who had an alcohol-related injury, the median BAC was 12 (IQR = 7–16; range 10 to 360) mg/dL.

Table 3 shows summary results for the first three questions of AUDIT (AUDIT-C). Patients who were BAC positive were significantly more likely to drink on a weekly basis, drink more in each drinking occasion, and binge drink on a monthly basis or more frequently. Of those who were BAC positive, 86% drank five drinks or more on a typical drinking occasion.

**Table 3:** AUDIT scores and AUDIT-C questions, *n* (%)

Measure	Category	Total (*n* = 883)	< 10 mg/dL (*n* = 755)	≥ 10 mg/dL (*n* = 128)	*X*^2^	*p*-value
AUDIT score, median^a^ (IQR)	–	8 (4–14)	6 (4–9)	15 (11–19)	–	< *0.001*
AUDIT categories	0–7	666 (75.5)	652 (86.3)	14 (11.0)		< *0.001*
	8–15	146 (16.5)	88 (11.7)	58 (45.3)		
	16–19	37 (4.2)	5 (0.7)	32 (25.0)		
	≥ 20	34 (3.9)	10 (1.3)	24 (18.8)		
Frequency	Never	502 (56.9)	500 (6.2)	2 (1.6)	398.5	< *0.001*
	Monthly or less	181 (20.5)	159 (21.1)	22 (17.2)		
	2–4 times/month	96 (10.9)	60 (7.9)	36 (28.1)		
	2–3 times/week	66 (7.5)	28 (3.7)	38 (29.7)		
	** ≥ **4 times/week	38 (4.3)	8 (1.1)	30 (23.4)		
Quantity	0–2	526 (59.6)	522 (69.1)	4 (3.1)	49.7	< *0.001*
	3–4	95 (10.8)	79 (10.5)	16 (12.5)		
	5–6	172 (19.5)	115 (15.2)	57 (44.5)		
	7–9	59 (6.7)	28 (3.7)	31 (24.2)		
	** ≥ **10	31 (3.5)	11 (1.5)	20 (15.6)		
Binge drinking	Never	654 (74.1)	618 (81.9)	36 (28.1)	55.0	< *0.001*
	Less than monthly	121 (13.7)	90 (11.9)	31 (24.2)		
	Monthly	40 (4.5)	23 (3.0)	17 (13.3)		
	Weekly	43 (4.9)	20 (2.6)	23 (18.0)		
	Daily or almost daily	25 (2.8)	4 (0.5)	21 (16.4)		

^a^Mean AUDIT score among drinkers (*n* = 396), differences between groups tested with Mann Whitney U test

### Injury profile

Table 4 shows that there was no significant difference in activity at the time of the injury or place it occurred for those alcohol-related or not. There was a significant difference in mechanism where more alcohol related injuries were transport-related (35.9% vs 27.2%, *p* < 0.001) and assault (29.7% vs 15.8%, *p* < 0.001). Alcohol-related injuries were commonly intentional or deliberate self-harm; intentional injuries accounted for 30.5% of injuries among those with positive BAC compared to 15.4% of those who were negative.

**Table 4:** Injury profile by alcohol-related status, *n* (%)

Characteristic	Category	Total (*n* = 883)	< 10 mg/dL (*n* = 755)	≥ 10 mg/dL (*n* = 128)	*X*^2^	*p*-value
Mechanism	Transport	251 (28.4)	205 (27.2)	46 (35.9)	32.0	< *0.001*
	Mechanical	180 (20.4)	163 (21.6)	17 (13.3)		
	Animal or plant bite/sting	161 (18.2)	153 (20.3)	8 (6.3)		
	Assault	157 (17.8)	119 (15.8)	38 (29.7)		
	Fall	69 (7.8)	58 (7.7)	11 (8.6)		
	Poisoning	45 (5.1)	38 (5.0)	7 (5.5)		
	Other^a^	20 (2.3)	19 (2.5)	1 (0.8)		
Place	Street/road/highway	339 (38.4)	280 (37.1)	59 (46.1)	12.5	*0.029*
	Home	312 (35.3)	276 (36.6)	36 (28.1)		
	Occupational	155 (17.6)	140 (18.5)	15 (11.7)		
	Other^b^	49 (5.5)	37 (4.9)	12 (9.4)		
	Leisure, recreation, sport	14 (1.6)	11 (1.5)	3 (2.3)		
	Public administrative area	14 (1.6)	11 (1.5)	3 (2.3)		
Activity	Travelling	323 (36.6)	264 (35.0)	59 (46.1)	8.3	0.082
	Vital activities	238 (27.0)	209 (27.7)	29 (22.7)		
	Working	168 (19.0)	152 (20.1)	16 (12.5)		
	Other^c^	119 (13.5)	100 (13.2)	19 (14.8)		
	Sport or leisure	35 (4.0)	30 (4.0)	5 (3.9)		
Intent	Unintentional	683 (77.3)	602 (79.7)	81 (63.3)	18.4	< *0.001*
	Intentional	155 (17.6)	116 (15.4)	39 (30.5)		
	Deliberate self-harm	45 (5.1)	37 (4.9)	8 (6.3)		

^a^Includes: ‘Other’, unknown, blast injuries, firearm, foreign body, other, exposure to electric current/heat/hot substances/smoke/fire/flame, drowning, animal/plan stings/bites/attacks, poisoning, fall; ^b^Includes: ‘other’, data not available/unknown, educational institution, leisure or recreational area/sport

^c^Includes: ‘other’, unknown/data not available, leisure activities, whilst in educational activities, doing sport

Data were available for 871 patients on their view of whether or not their injury was preventable, to which 17.6% responded the injury was preventable and 4.9% responded that the injury would have been preventable if alcohol had not been used (directly or indirectly). A significantly higher proportion of alcohol-related injuries than non-alcohol-related injuries were believed to be preventable if alcohol had not been consumed (28.1% vs 0.9%, respectively, *p* < 0.001).

### Factors associated with an alcohol-related injury

We conducted logistic regression for alcohol-related injuries to assess which variables were associated with an increased risk of having an alcohol-related injury, including injury mechanism, occupation, age, and injury intent in the final model (Table 5). The model was significant and explained 20% of the variance in alcohol-related injuries and correctly classified 85.5% of cases. The analysis showed that compared to animal/plant bites/stings, transport injuries were more likely to be alcohol-related (OR = 5.14, 95% CI: 2.30, 11.49) as were falls (OR = 4.99, 95%CI: 1.84, 13.53). Alcohol-related injuries were more likely to be intentional than unintentional (OR = 3.47, 95% CI, 1.01, 11.87) but there was no significant association with deliberate-self harm. Of note, although self-poisoning was the most common form of deliberate self-harm (40/45, 88.9%), only six (15.0%) were BAC positive. Compared to the age group 18–25 years, there was a higher odds ratio of an alcohol-related injury among 26–40-year-olds (OR = 2.29, 95% CI:1.11, 4.72) and 41–55-year-olds (OR = 2.76, 95% CI: 1.29, 5.90). There were no significantly increased odds for an alcohol-related injury depending on occupation group, but compared to salaried employed the odds of an alcohol-related injury was significantly lower (OR = 0.37, 95% CI:0.16, 0.81).

**Table 5:** Factors associated with an alcohol-related injury in univariate and multivariate binary logistic regression

		**Unadjusted**			**Adjusted**		
**Variable**	**Category**				**OR**	**95% CI**	***p*-value**
Mechanism	Animal/plant sting/bite	ref			ref		
	Transport	4.29	(1.97, 9.36)	< 0.001	5.14	(2.30, 11.49)	** < 0.001**
	Assault	6.11	(2.75, 13.58)	< 0.001	2.29	(0.55, 9.60)	0.257
	Mechanical	2.00	(0.34, 4.76)	0.119	1.63	(0.66, 4.03)	0.290
	Poisoning	3.52	(1.20, 10.32)	0.022	0.35	(0.16, 11.20)	0.783
	Fall	6.33	(1.39, 9.47)	0.008	4.99	(1.84, 13.53)	**0.002**
	Other	1.01	(0.12, 8.50)	0.995	1.01	(0.11, 9.14)	0.993
Intent	Unintentional	ref			ref		
	Intentional	2.50	(1.62, 3.84)	< 0.001	3.47	(1.01, 11.87)	**0.047**
	Deliberate self-harm	1.61	(0.72, 3.57)	0.244	5.73	(0.84, 39.00)	0.074
Occupation	Salaried employed	ref			ref		
	Farmer	1.69	(0.83, 3.44)	0.150	1.85	(0.84, 4.09)	0.127
	Self-employed	1.59	(0.64, 3.96)	0.322	1.67	(0.64, 4.34)	0.291
	Daily wage labour	2.34	(1.12, 4.88)	0.024	1.99	(0.91, 4.35)	0.084
	Other	0.44	(0.20, 9.34)	0.033	0.37	(0.16, 0.81)	**0.014**
Age group	18–25	ref			ref		
	26–40	2.32	(1.17, 4.57)	0.016	2.29	(1.11, 4.72)	**0.025**
	41–55	3.10	(1.55, 6.17)	0.001	2.76	(1.29, 5.90)	**0.009**
	≥ 55	1.74	(0.78, 3.88)	0.180	1.69	(0.68, 4.18)	0.258

Hosmer and Lemeshow: X^2^(8) = 5.584, *p* = 0.664, Nagelkerke *R* = 0.197

## Discussion

This study reports the prevalence of alcohol-related injury in an inpatient-hospital sample across three hospitals in the North-Central Province in Sri Lanka. We found that a considerable amount of injuries in this sample were alcohol-related; 15% of all injury cases and 32.8% among current drinkers (individuals who have used alcohol over the last 12 months). Patients with alcohol-related injury were almost exclusively male and aged 41–55 years. Injuries that were most commonly associated with acute alcohol use were traffic-related and likely to be intentional.

The prevalence of abstinence in the last 12 months in this sample of injured people (55.2%) is considerably lower than WHO data for Sri Lanka which show that 71.3% of men and women abstained in the last 12 months [1]. This may suggest that alcohol use in this region may be higher than the national average or that people with injury are more likely to use alcohol. Major efforts to address alcohol use and subsequent harm is needed for both this region and Sri Lanka. Reducing current alcohol use is already part of the 2016–20 national prevention strategy for noncommunicable diseases (NCDs), with particular focus on illicit alcohol and reducing alcohol-related injury [23]. A new NCD strategy may need to focus on reducing alcohol consumption to meet the global target of a 10% reduction in alcohol use by 2025 [24].

The prevalence of alcohol-related injuries in the current study should be considered within the context of wider alcohol policy and efforts to implement effective policy measures to address alcohol-related harm. A study including ED injury data from 28 different countries indicated that the overall strength of alcohol policy was a strong predictor of the incidence of alcohol-related injury [25]. The same study also reported a high prevalence of alcohol use within the six hours before injury in India, with 78.6% of current drinkers (any alcohol in the last 12 months) reporting such, although only 25.7% of the sample were defined as current drinkers compared to 44.8% in our sample across three hospitals in one region in Sri Lanka [25]. While our work shows a high prevalence of alcohol-related injury among a sample of current drinkers, future research should explore whether the prevalence is similar in other rural areas, and how and what aspects of alcohol policy may influence these rates of alcohol-related injury.

Drinking patterns and some alcohol-related policy measures, such as drink-driving, appear to be related to level of alcohol-related injury. Cherpitel and colleagues [18] found that higher overall consumption and detrimental drinking patterns were found in countries with higher legal BAC limit for driving and in these settings there was also a higher prevalence of alcohol-related injuries. A recent study shows that Sri Lanka, along with other South-East Asian countries, have made some efforts to strengthen alcohol policies between 2010 and 2017 [26]. However, it is possible that policies such as drink-driving are in place but not adhered to in rural settings or adequately enforced. The *Global status report on road safety* in 2018 reported that the enforcement level score of drink-driving laws in Sri Lanka was rated as 9/10, however it reported that random breath testing is not implemented and the legal BAC of 80 mg/dL is higher than WHO best practice (50 mg/dL for all drivers and 20 mg/dL for young/novice drivers) [27]. Further work is needed to explore how these policies are enforced as road injury has been estimated to be the category of alcohol-related harm with the highest economic impact [2]. Effective enforcement of drink-driving initiatives could therefore have a positive impact on preventing injury and mortality, as well as reducing economic costs. In the current study, we have shown that a higher proportion of transport injuries were alcohol-related (35.9% vs 27.2%) but not the context in which they occurred (driver or road user). However, only 18% of all transport injuries were alcohol-related and further is needed work to better understand these types of injuries and their relation to alcohol also in the context of being involved in road traffic accidents while intoxicated while for example walking.

Our findings also indicate that current drinking patterns are an important factor related to alcohol-related injury, as those with positive breathalyzer readings were significantly less likely to be abstainers or low risk drinkers. With about one in five of alcohol-related injury patients scoring positive for possible dependence, interventions are needed to address problem drinking in the communities in this region. The group sustaining alcohol-related injuries were male and significantly more likely to be aged between 26 and 55 years, with higher proportion working in farming and daily wage labor. Qualitative research in the same region found differences in alcohol use among younger and older men, with young men appearing to adopt more urban and Westernised behaviors and sought out more expensive, commercially produced alcohol which formed part of perceptions around social status [7]. Older men, on the other hand, preferred cheap and illicitly produced Kassipu which was considered by younger men to be a “marker of low social status” and associated with high/problematic drinking [7]. When considering the impact of alcohol on communities in this region, social structures and the practices of self-harm are vital considerations. A qualitative study including 24 individuals with experience of non-fatal self-poisoning found that a third of the participants had an alcohol use disorder (as defined in ICD-10), all of whom were male [28], and the impact of alcohol on self-harm has been captured previously [3].

### Limitations

The major limitation of our study was the limited number of hospitals we were able to include. In addition, there was incomplete information in the hospital records regarding the time of the injury and resource limitations meant we were unable to interview all patients who presented with an injury to ascertain their alcohol status. There were differences noted in the sample between people who had a breath sample versus those without. Those without a breath sample were likely to be older and female. This group is also more likely to be abstainers as drinking is not common across Sri Lanka in older females. A recent study showed only 1.2% of women were current drinkers. Therefore, we think that while the people without a breathalyser sample were somewhat different that the people with significant impairment were most likely to be captured by the survey.

The use of BAC as the indicator for an alcohol-related injury may have missed cases where alcohol was consumed prior to the injury but who will have been negative at the time of admission due to time elapsed between the events. However, we used a low BAC threshold to define recent alcohol use; this would have allowed us to identify patients with long intervals to sampling who had drunk relatively large amounts of alcohol. Further, we did not find any significant difference between patients where time of injury was known compared to unknown, so underestimation of positive BAC in this group did not appear to be an issue. Previous studies have used cut-off time of six hours between injury and admissions [18] but we were unable to apply this criteria to our findings. Thus, the results are likely to be an under-representation of the role of acute alcohol in injury. As only two individuals who provided a positive breathalyser sample reported being non-drinkers, it may suggest that reporting of current drinking may be an accurate account which strengthens the findings regarding current alcohol use in this group. As this study focused on in-patients, and therefore more severe injury cases, the involvement of alcohol may have been greater than an outpatient or community sample. Finally, this study was conducted in three hospitals in one of the nine regions in Sri Lanka. As such, these findings represent the burden of hospital admissions for injury and may not be generalisable to other parts of the country.

## Conclusions

These findings of injury commonly associated with alcohol consumption and hazardous use highlight the need for a systemic approach to reducing and preventing alcohol-related harm. Of particular focus is the need for exploring the role of alcohol among working age men and the socio-cultural role of alcohol in these communities. Injury prevention efforts that target road safety as well as interpersonal violence need to incorporate the role that alcohol play in these injuries.

## Supplementary Information

**Additional file 1.**

**Additional file 2.**

## Data Availability

The datasets generated and/or analysed during the current study are not publicly available due to that it may compromise individual privacy but are available from the corresponding author on reasonable request. This is in line with our ethical approval.

## References

[1] WHO. Global status report on alcohol health. Geneva: World Health Organization; 2018. https://www.who.int/publications/i/item/9789241565639. Accessed 15 Mar 2022.

[2] Ranaweera S, Amarasinghe H, Chandraratne N, Thavorncharoensap M, Ranasinghe T, Karunaratna S (2018). Economic costs of alcohol use in Sri Lanka. PLoS ONE.

[3] Sørensen JB, Agampodi T, Sørensen BR, Siribaddana S, Konradsen F, Rheinländer T (2017). “We lost because of his drunkenness”: The social processes linking alcohol use to self-harm in the context of daily life stress in marriages and intimate relationships in rural Sri Lanka. BMJ Glob Health.

[4] Katulanda P, Ranasinghe C, Rathnapala A, Karunaratne N, Sheriff R, Matthews D (2014). Prevalence, patterns and correlates of alcohol consumption and its’ association with tobacco smoking among Sri Lankan adults: A cross-sectional study. BMC Public Health.

[5] Rehm J, Kanteres F, Lachenmeier D (2010). Unrecorded consumption, quality of alcohol and health consequences. Drug Alcohol Rev.

[6] Babor TF, Caetano R, Casswell S, Edwards G, Giesbrecht N, Graham K, et al. Alcohol: no ordinary commodity: research and public policy. 2nd ed. Oxford: Oxford University Press; 2010.

[7] Sørensen JB, Konradsen F, Agampodi T, Sørensen BR, Pearson M, Siribaddana S (2020). A qualitative exploration of rural and semi-urban Sri Lankan men’s alcohol consumption. Glob Public Health.

[8] Jayasinghe NRM, Foster JH (2011). Deliberate self-harm/ poisoning, suicide trends. The link to increased alcohol consumption in Sri Lanka. Archives of Suicide Research.

[9] Knipe DW, Gunnell D, Pearson M, Jayamanne S, Pieris R, Priyadarshana C (2018). Attempted suicide in Sri Lanka – An epidemiological study of household and community factors. J Affect Disord.

[10] Damayanthi HDWT, Moy FM, Abdullah KL, Dharmaratne SD (2018). Prevalence of malnutrition and associated factors among community-dwelling older persons in Sri Lanka: A cross-sectional study. BMC Geriatr.

[11] Lakmal MAC, Ekanayake EMDNK, Kelum SHP, Gamage BD, Jayasundara JASB. Hospital-Based Case Series Analysis of Road Traffic Trauma Patients in Sri Lanka. Indian J Surg. 2020;17(83):1–6. 10.1007/s12262-020-02473-8. 10.1007/s12262-020-02473-8 PMC7298447 32837075

[12] Navaratne KV, Fonseka P, Rajapakshe L, Somatunga L, Ameratunga S, Ivers R, et al. Population-based estimates of injuries in Sri Lanka. Inj Prev. 2009;15:170–5. 10.1136/ip.2008.019943 19494096

[13] Samarasinghe D (2006). Sri Lanka: alcohol now and then. Addiction.

[14] Rehm J, Gmel G, Sempos CT, Trevisan M (2003). Alcohol-related morbidity and mortality. Alcohol Res Health.

[15] Taylor B, Irving HM, Kanteres F, Room R, Borges G, Cherpitel C, Rehm J (2010). The more you drink the harder you fall: A systematic review and meta-analysis of how acute alcohol consumption and injury or collission risk increase together. Drug Alcohol Depend.

[16] Rehm J, Monteiro M, Room R, Gmel G, Jernigan D, Frick U (2001). Steps towards constructing a global comparative risk analysis for alcohol consumption: determining indicators and empirical weights for patterns of drinking, deciding about theoretical minimum, and dealing with different consequences. Eur Addict Res.

[17] Cherpitel CJ, Witbrodt J, Korcha RA, Ye Y, Monteiro MG, Chou P (2019). Dose–response relationship of alcohol and injury cause: effects of country-level drinking pattern and alcohol policy. Alcoholism: Clinical and Experimental Research.

[18] Cherpitel CJ, Ye Y, Bond J, Rehm J, Poznyak V, Macdonald S (2005). Multi-level analysis of alcohol-related injury among emergency department patients: A cross-national study. Addiction.

[19] Chou SP, Chun S, Smith S, Ruan J, Li TK, Grant BF (2012). Episodic heavy drinking, problem drinking and injuries - Results of the WHO/NIAAA collaborative emergency room study in South Korea. Alcohol.

[20] Cherpitel CJ, Bond J, Ye Y, Borges G, Macdonald S, Giesbrecht N (2003). A cross-national meta-analysis of alcohol and injury: Data from the Emergency Room Collaborative Alcohol Analysis Project (ERCAAP). Addiction.

[21] De Silva P, Jayawardana P, Pathmeswaran A (2008). Concurrent validity of the alcohol use disorders identification test (AUDIT). Alcohol Alcohol.

[22] de Silva P, Jayawardana P, Pathmeswaran A. Prevalence of and risk factors for hazardous drinking and alcohol use disorders among married men in Wattala Divisional Secretariat area, thesis for MD [Community Medicine]. 2006.

[23] Ministry of Health Nutrition and Indigenous Medicine Sri Lanka. National Multisectoral Action Plan for the Prevention and Control of Noncommunicable diseases 2016–2020. 2017. https://www.iccp-portal.org/system/files/plans/national_ncd_action_plan_sri_lanka.pdf.

[24] WHO. Global NCD target: Reduce the harmful use of alcohol. Geneva: World Health Organization; 2016. https://www.who.int/beat-ncds/take-action/policy-brief-reduce-alcohol.pdf. Accessed 15 Mar 2022.

[25] Cherpitel CJ, Witbrodt J, Korcha RA, Ye Y, Kool B, Monteiro M (2018). Multi-level analysis of alcohol-related injury, societal drinking pattern and alcohol control policy: emergency department data from 28 countries. Addiction.

[26] Sornpaisarn B, Shield K, Manthey J, Limmade Y, Low WY, van Thang V (2020). Alcohol consumption and attributable harm in middle-income South-East Asian countries: Epidemiology and policy options. International Journal of Drug Policy..

[27] WHO. Global status report on road safety 2018. Geneva: World Health Organization; 2018. https://www.who.int/publications/i/item/9789241565684. Accessed 15 Mar 2022.

[28] Rajapakse T, Griffiths KM, Christensen H, Cotton S. Non-fatal self-poisoning in Sri Lanka: Associated triggers and motivations. BMC Public Health. 2015;15:1167. 10.1186/s12889-015-2435-5. 10.1186/s12889-015-2435-5 PMC4659153 26602540

[29] Schölin L, Agampodi S, Charhurang U, Weerasinghe M, Holloway A, Norrie J, et al. Prevalence of alcohol-related injury across three hospitals in rural Sri Lanka. Conference paper presented at the Asia-Pacific Academic Consortium for Public Health, 5-9 December 2020. http://www.slma.lk/abstracts/presentations1.html. Accessed 6 Dec 2021.

