# Supplementary figures and images for: Involvement of alcohol in injury cases in rural Sri Lanka: prevalence and associated factors among in-patients in three primary care hospitals
Source: BMC Public Health. 2022 Mar 16;22:514. doi: 10.1186/s12889-022-12958-8 (PMC8928674; doi:10.1186/s12889-022-12958-8)

**SUPPLEMENTARY FILE 1 – INJURY DATA COLLECTION FORM**


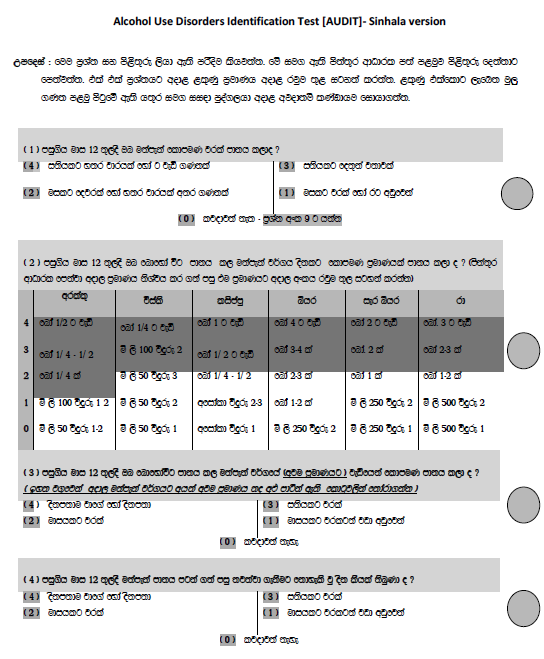
**SUPPLEMENTARY FILE 2 – AUDIT (SINHALA VERSION)**


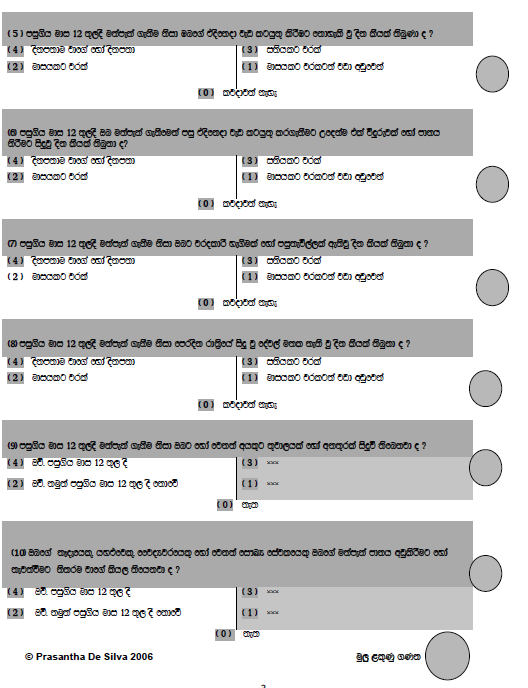

Supplement: Supplementary file 1 — Additional file 1. [file 12889_2022_12958_MOESM1_ESM.docx]
