# Supplementary material for: Involvement of alcohol in injury cases in rural Sri Lanka: prevalence and associated factors among in-patients in three primary care hospitals
Source: BMC Public Health. 2022 Mar 16;22:514. doi: 10.1186/s12889-022-12958-8 (PMC8928674; doi:10.1186/s12889-022-12958-8)
Supplement: Supplementary file 2 — Additional file 2. [file 12889_2022_12958_MOESM2_ESM.pdf]

The adapted Alcohol Use Disorders Identification Test [AUDIT] for Sri Lanka – English Version

Brief introduction

The Alcohol Use Disorders Identification Test (AUDIT) is a widely used screening instrument to detect early stages of at-risk drinking patterns for acute and chronic alcohol-related problems and harmful use and alcohol dependence. The first three question items of the AUDIT examine the quantity and frequency of ethanol consumption and its patterns; the rest focuses on the consequences of harmful use and key dependence symptoms.

The maximum possible scoring is limited to 40.

The interpretation key:

The key to interpretation of the total scores obtained from the question items is as follows,

- **1 – 7 = Low-Risk Drinking**
- **8 – 15 = Hazardous Drinking**
- **16 – 19 = Probable Harmful Use**
- **20 or more = Probable Dependence**

Since the AUDIT is a screening instrument, it cannot be used for diagnostic purposes.

Citation

I adapted and validated the above instrument using community and hospital samples as a part of my thesis for the MD [Community Medicine] examination held by the Post-Graduate Institute of Medicine of the University of Colombo, Sri Lanka, in 2006. Prof. Pushpa Jayawardana and Prof. A. Pathmeswaran supervised the study. Those who expect to use this instrument are requested to acknowledge and write the citation as follows;

De Silva Prasantha, Jayawardana P, Pathmeswaran A [2006] Prevalence of and risk factors for hazardous drinking and alcohol use disorders among married men in Wattala Divisional Secretariat area, thesis for MD [Community Medicine], June, 2006.

For further information, contact- prasantha.silva@gmail.com

The adapted Alcohol Use Disorders Identification Test [AUDIT] for Sri Lanka – English version

Interviewer instructions: Read as it is written here.

"Now I am going to ask some questions about your use of alcoholic beverages during past 12 months from now on. The alcoholic beverages here refer to types of beers such as Larger, Carlsberg, Byson, Arracks such as Gal, Pol, Old, Toddy and Kassippu (Suduwa)". Show the visual aids. Do not discriminate Kassipu (the unrecorded product) from the legal products. Find the risk category by adding all points for the ten questions.

(1) **During past 12 months how often did you have an alcoholic drink?**

**(4)**	**4 or more times a week**	**(3)**	**2-3 times a week**
**(2)**	**Once a week or once in two weeks**	**(1)**	**Once a month or fewer**
		**(0)**	**Never (Skip to 9-10 questions)**

(2) **During past 12 months how many drinks of your ------ (show the visual aid) did you have on a typical day?** (Choose the relevant amount and the score from the following table)

	Arrack	Whisky	Kassippu	Beer	Strong beer	Toddy
**(4)**	**>1/2bottle**	**>1/4 bottle**	**>1 bottle**	**>4 bottles**	**>2 bottles**	**>3 bottles**
**(3)**	**¼-1/2 bottles**	**2 glasses of 100ml**	**>1/2 bottle**	**3-4 bottles**	**2 bottles**	**2-3 bottles**
**(2)**	**¼ bottle**	**3 glasses of 50ml**	**¼-1/2 bottles**	**2-3 bottles**	**1 bottle**	**1-2 bottles**
**(1)**	**1-2 glasses of 100ml**	**2 glasses of 50 ml**	**2-3 Asoka glasses**	**1-2 bottles**	**2 glasses of 250ml**	**2 glasses of 500ml**
**(0)**	**1-2 glasses of 50ml**	**1 glass of 50ml**	**1 glass of Asoka glass**	**2 glasses of 250ml**	**1 glass of 250ml**	**1 glass of 500ml**

(3) **<u>During past 12 months, how often did you have</u>** <u>(choose the relevant amount of the respondent's favorite brand in glasses or bottles cited in the colored strip from the above table)</u> **<u>or more in your usual drinking day?</u>**

**(4)**	**Daily or almost daily**	**(3)**	**Once a week**
**(2)**	**Once a month**	**(1)**	**Fewer than a month**
	**(0)**	**Never**	

(4) **<u>During past 12 months how often were you unable to stop drinking once you started?</u>**

**(4)**	**Daily or almost daily**	**(3)**	**Once a week**
**(2)**	**Once a month**	**(1)**	**Fewer than a month**
	**(0)**	**Never**	

(5) **<u>During past 12 months how often have you failed to do what was normally expected from you because of drinking?</u>**

**(4)**	**Daily or almost daily**	**(3)**	**Once a week**
**(2)**	**Once a month**	**(1)**	**Fewer than a month**
	**(0)**	**Never**	

(6) **<u>During past 12 months how often have you needed a first drink in the morning to get yourself going after a heavy drinking session?</u>**

**(4)**	**Daily or almost daily**	**(3)**	**Once a week**
**(2)**	**Once a month**	**(1)**	**Fewer than a month**
	**(0)**	**Never**	

(7) <u>During past 12 months how often have you had a feeling of guilt or remorse after drinking?</u>

**(4) Daily or almost daily**	**(3) Once a week**
**(2) Once a month**	**(1) Fewer than a month**
**(0) Never**	

(8) <u>During past 12 months how often have you been unable to remember what happened the night before because of drinking?</u>

**(4) Daily or almost daily**	**(3) Once a week**
**(2) Once a month**	**(1) Fewer than a month**
**(0) Never**	

(9) <u>Have you or someone else been injured as a result of your drinking?</u>

**(2) Yes, during the past 12 months**	**(1) Yes, but not during the past 12 months**
**(0) Never**	

(10) <u>Has a relative or a friend or a doctor or another health worker or anyone been concerned about your drinking and suggested that you cut down on drinking?</u>

**(2) Yes, during the past 12 months**	**(1) Yes, but not during the past 12 months**
**(0) Never**	

Total score

The visual aid

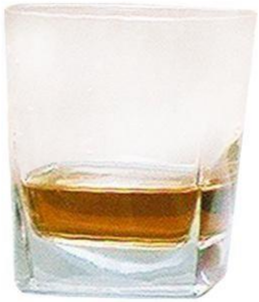

50 ml

Whisky

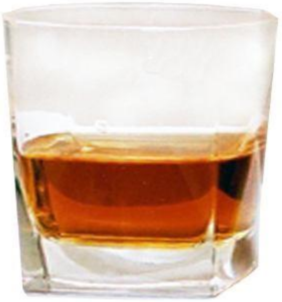

100 ml

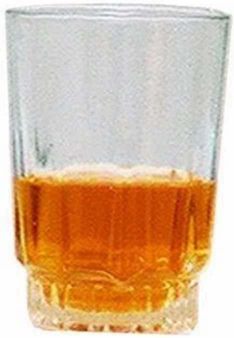

50ml

Arrack

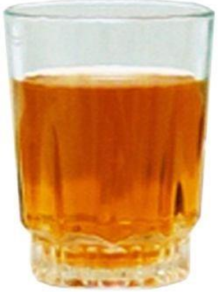

100 ml

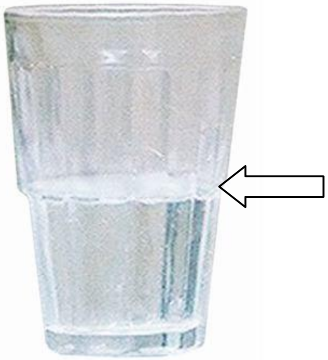

50ml

Kassippu

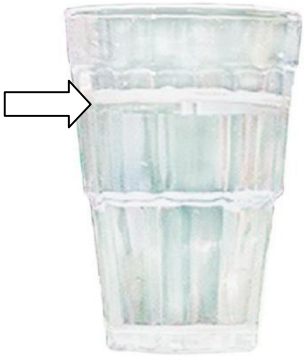

100 ml

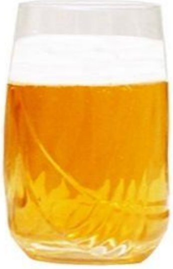

Beer 250 ml glass

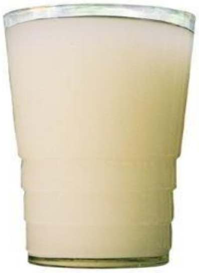

Toddy 500 ml glass

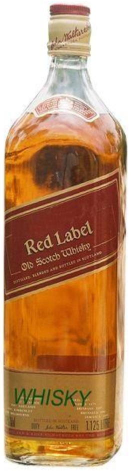

Whisky

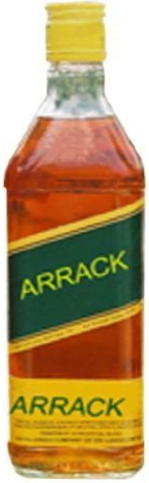

Arrack

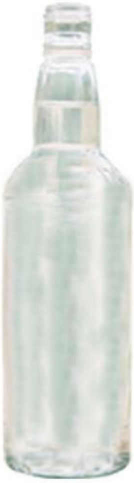

Kassippu

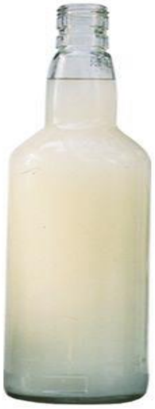

Toddy

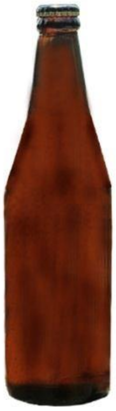

Strong beer

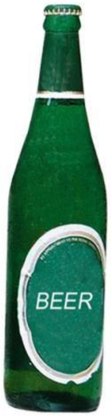

Normal beer

© Prasantha De Silva
